# Supplementary material for: Analysis of Infectious Complications after Thermal Ablation of Hepatocellular Carcinoma and the Impact on Long-Term Survival
Source: Cancers (Basel). 2022 Oct 23;14(21):5198. doi: 10.3390/cancers14215198 (PMC9657620; doi:10.3390/cancers14215198)
Supplement: Supplementary file 1 [file cancers-14-05198-s001.zip › cancers-1950026-supplementary.pdf]

Supplemental Data

Table S1 Baseline characteristics of infection in mild and severe patients

	Mild Infection (41 patients)	Severe Infection (8 patients)	P Value
Age (years)	58.2±13.0	63.8±10.9	0.267
Gender (M/F)	85.4%(35/6)	100.0%(8/0)	0.571
Hepatic Cirrhosis(Y/N)	85.4%(35/6)	100.0%(8/0)	0.571
Diabetes(Y/N)	29.3%(12/29)	12.5%(1/7)	0.586
HBsAg (+)(Y/N)	87.8%(36/5)	87.5%(7/1)	1.000
Child-Pugh class			1.000
A	92.7%(38)	100%(8)	
B	7.3%(3)	0%(0)	
Alanine aminotransferase level(U/L)	36.9±28.3	58.9±71.2	0.416
Aspartate aminotransferas Level(U/L)	47.6±55.3	62.6±52.2	0.483
Total bilirubin Level(mg/dL)	18.5±9.3	35.1±50.2	0.382
Alpha- fetoprotein Level(ug/L)	1562.0±6887.2	1337.2±2902.0	0.929
Thermal ablation type			1.000
Radiofrequency ablation	92.7%(38)	87.5%(7)	
Microwave ablation	7.3%(3)	12.5%(1)	
Tumor number	1.9±0.9	1.9±0.9	0.939
Tumor size(maximum diameter)	2.4±1.1	1.9±0.6	0.212
Transcatheter arterial chemoembolization(Y/N)	43.9%(18/23)	12.5%(1/7)	0.204

M: male; F: female. Y: yes; N: no. Data are expressed as mean ± s.d.

Table S2 Univariate and multivariate COX regression analysis for OS.

Characteristics	Univariate analysis		Multivariate analysis	
	HR(95%CI)	P value	HR(95%CI)	P value
Age	1.717(0.881-3.348)	0.113		
< 60 yr				
≥ 60 yr				
Gender	1.934(0.462-8.098)	0.367		
Male				
Female				
Hepatic Cirrhosis	1.202(0.367-3.930)	0.761	0.467(0.125-1.743)	0.257
No				
Yes				
HBsAg (+)	0.290(0.350-1.685)	0.425		
No				
Yes				
Child-Pugh class	3.179(1.100-9.185)	0.033*	4.317(1.425-13.078)	0.010*
A				
B				
Diabetes	0.992(0.448-2.393)	0.984	1.064(0.475-2.383)	0.880
No				
Yes				
Alpha-fetoprotein (ug/L)	3.633(1.774-7.441)	0.000*	4.746(2.118-10.632)	0.000*
< 20				
≥ 20				
Thermal ablation type	0.584(0.140-2.44)	0.461		
Radiofrequency ablation				
Microwave ablation				
Postoperative infection	1.356(0.697-2.637)	0.369	1.410(0.719-2.766)	0.318
No				
Yes				

*Significant difference.
